# Opportunistic Mapping of *Strongyloides stercoralis* and Hookworm in Dogs in Remote Australian Communities

**DOI:** 10.3390/pathogens9050398

**Published:** 2020-05-21

**Authors:** Meruyert Beknazarova, Harriet Whiley, Rebecca Traub, Kirstin Ross

**Affiliations:** 1 Faculty of Science and Engineering, Flinders University, Bedford Park, SA 5042, Australia; harriet.whiley@flinders.edu.au (H.W.); kirstin.ross@flinders.edu.au (K.R.); 2 Faculty of Veterinary and Agricultural Sciences, University of Melbourne, Parkville, VIC 3052, Australia; rebecca.traub@unimelb.edu.au

**Keywords:** *Strongyloides stercoralis*, soil-transmitted helminths, hookworms, zoonotic parasites, Australian remote communities, One Health

## Abstract

Both *Strongyloides stercoralis* and hookworms are common soil-transmitted helminths in remote Australian communities. In addition to infecting humans, *S. stercoralis* and some species of hookworms infect canids and therefore present both environmental and zoonotic sources of transmission to humans. Currently, there is limited information available on the prevalence of hookworms and *S. stercoralis* infections in dogs living in communities across the Northern Territory in Australia. In this study, 274 dog faecal samples and 11 faecal samples of unknown origin were collected from the environment and directly from animals across 27 remote communities in Northern and Central Australia. Samples were examined using real-time polymerase chain reaction (PCR) analysis for the presence of *S. stercoralis* and four hookworm species: *Ancylostoma caninum, Ancylostoma ceylanicum, Ancylostoma braziliense* and *Uncinaria stenocephala*. The prevalence of *S. stercoralis* in dogs was found to be 21.9% (60/274). *A. caninum* was the only hookworm detected in the dog samples, with a prevalence of 31.4% (86/274). This study provides an insight into the prevalence of *S. stercoralis* and hookworms in dogs and informs future intervention and prevention strategies aimed at controlling these parasites in both dogs and humans. A “One Health” approach is crucial for the prevention of these diseases in Australia.

## 1. Introduction

Soil-transmitted helminths (STHs) are estimated to infect up to 2 billion people worldwide, with a high prevalence recorded in Southeast Asia [1,2,3]. Australia as a whole has a relatively low prevalence of STHs due to widespread access to adequate hygiene, sanitation and clean water [4]. *Strongyloides stercoralis,* distributed throughout the tropics, is estimated to infect up to 370 million people worldwide, predominantly in socioeconomically disadvantaged communities [5,6]. Strongyloidiasis is a major health concern in remote Australian communities with up to 60% of indigenous populations found to be seropositive for the disease [4,7,8]. *Strongyloides stercoralis* can infect humans chronically and, in the case of immunocompromised patients, can develop into severe hyperinfective or disseminated strongyloidiasis, which has a mortality rate of up to 90% [9].

Genetic studies worldwide and in Australia have shown that there are at least two genetically different strains of *S. stercoralis*—one that is zoonotic, infecting both humans and dogs, and one that only infects dogs [10,11,12]. There is sufficient evidence to suggest that dogs can act as potential reservoirs for human strongyloidiasis and that controlling the parasite in dogs may play a role in preventing the disease in humans.

Hookworms infect up to half a billion people worldwide [13]. The most prevalent hookworms in humans in Southeast Asia and the Pacific are *Necator americanus, Ancylostoma ceylanicum*, and *Ancylostoma duodenale* [14,15]. Hookworms in humans can contribute to iron deficiency anaemia and can have an impact on maternal and child health [16]. Hookworm infection in humans was considered a widespread public health problem in parts of Australia until intervention campaigns successfully eradicated it from the mainstream population [17,18,19,20,21]. Only a single autochthonous case of *A. ceylanicum* in humans was reported in Western Australia and an imported case was reported in an Australian soldier returning from the Solomon Islands [22,23]. More recent studies found that hookworms, specifically *A. duodenale* [24], remain sporadically reported in remote communities in far north Queensland, northern parts of New South Wales, Western Australia and the Northern Territory (NT). In the Northern Territory, hookworm prevalence in humans is reported to be significantly lower than that of *S. stercoralis* [18,21,22,25]. Overall, a reduction has been seen in both *S. stercoralis* and hookworm infections in humans in the remote communities in the NT, and this has been attributed to deworming programs [20]. However, neither strongyloidiasis nor hookworm infection has been eradicated completely from remote communities, despite various intervention programs.

In Australia, as in other countries of the Asia-Pacific region, dogs are considered a potential zoonotic reservoir for STH infections, including strongyloidiasis and hookworms. Within indigenous Australian communities, the risk of transmission may be increased by the fact that dogs tend to live in close contact with humans [26].

In Australia, the most common hookworms in dogs are *Ancylostoma caninum*, *A. ceylanicum*, *Ancylostoma braziliense* and *Uncinaria stenocephala* [15]. These hookworm species are zoonotic and all are capable of causing cutaneous larva migrans in humans [27]. *A. ceylanicum* and *A. caninum* are of particular interest, as *A. ceylanicum* larvae can develop into the adult stage in humans, and *A. ceylanicum* is now recognised as the second most common species of hookworm infecting humans in the Asia-Pacific [28,29,30]. *A. caninum* infection in humans is non-patent and is strongly associated with eosinophilic enteritis [31,32]. Recent data show a high prevalence of both *A. caninum* and *A. ceylanicum* in dogs, dingoes and soil in remote communities in Western Australia and North-East Queensland. [33,34]. Both *A. ceylanicum* and *A. caninum* are considered neglected zoonotic parasites and accurate data on their prevalence in dogs and humans residing in the Indigenous communities of northern Australia are largely lacking [15,24,28,32,35]. 

In this study, we aimed to map the distribution of zoonotic *S. stercoralis* and hookworm species in dogs in remote communities in northern Australia. To the best of our knowledge, this is the first large-scale molecular study of dogs in these remote communities for the presence of *S. stercoralis* and hookworms.

## 2. Results

### 2.1. Dog DNA Origin

We tested 285 fresh faecal samples, presumed to be from dogs, which had been collected from communities across the Northern Territory, Central Australia, northern areas of Western Australia and the north-west of South Australia. These samples were screened for *Canis lupus familiaris* and *Canis lupus dingo* DNA. We confirmed that 274 out of 285 DNA samples extracted from the faeces were of dog origin (*Canis lupus familiaris* or *Canis lupus dingo*) through the use of polymerase chain reaction (PCR)-based amplification of the partial mitochondrial DNA (mtDNA).

### 2.2. Prevalence of *Strongyloides stercoralis* and Hookworms

**Figure 1:**
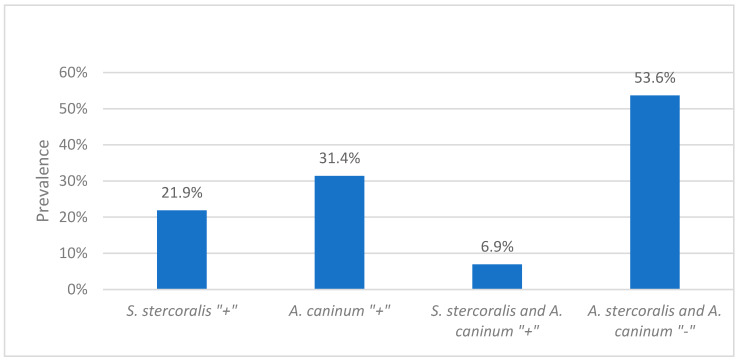
The percentage of dog faecal samples positive for *Strongyloides. stercoralis*, *Ancylostoma caninum, S. stercoralis* and *A. caninum* and the percentage of dog samples negative for both *S. stercoralis* and *A. caninum.*

The prevalence of *Strongyloides* species (spp.) among the 285 environmental faecal samples was 21.1% (60/285) as determined by PCR-based amplification of the partial 18 Svedberg unit ribosomal RNA (18S rRNA). The prevalence of *S. stercoralis* among the 274 dog faecal samples was 21.9% (60/274) (Figure 1).

Out of four hookworm species tested, only *A. caninum* was detected. The prevalence of hookworm infection (*A. caninum*) among the 285 environmental faecal samples was 30.2% (86/285) by PCR-based amplification of the partial internal transcribed spacer (ITS) gene. The prevalence of hookworm infection (*A. caninum*) among the 274 dog samples was 31.4% (86/274) (Figure 1).

**Figure 2:**
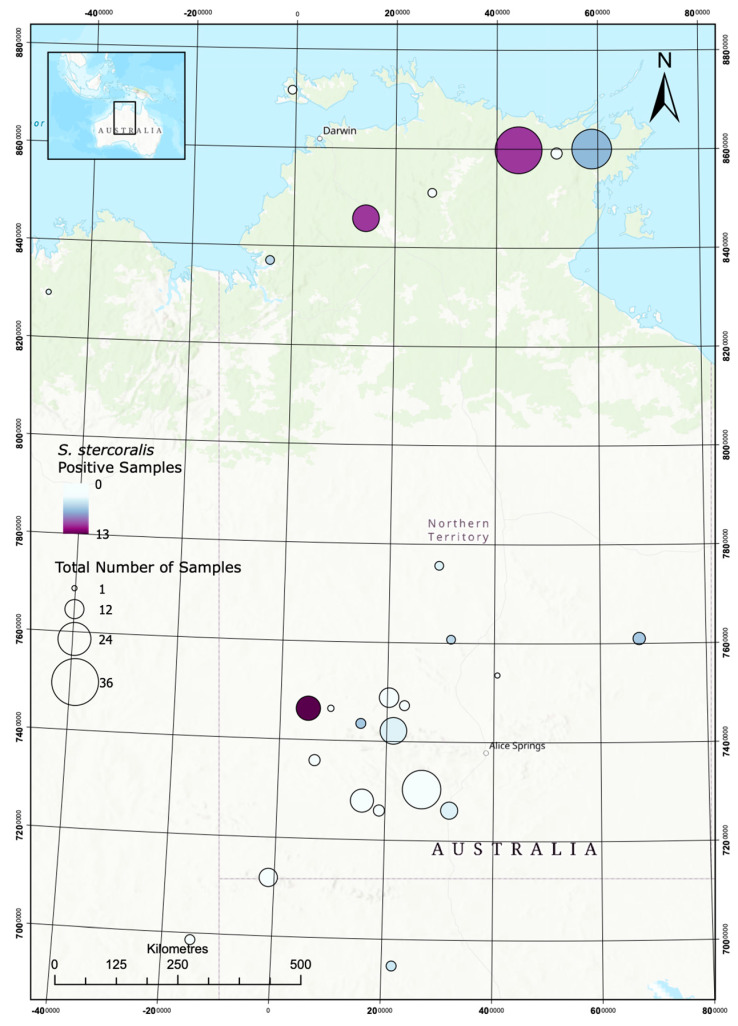
Opportunistic mapping of *S. stercoralis* in dogs in remote communities.

**Figure 3:**
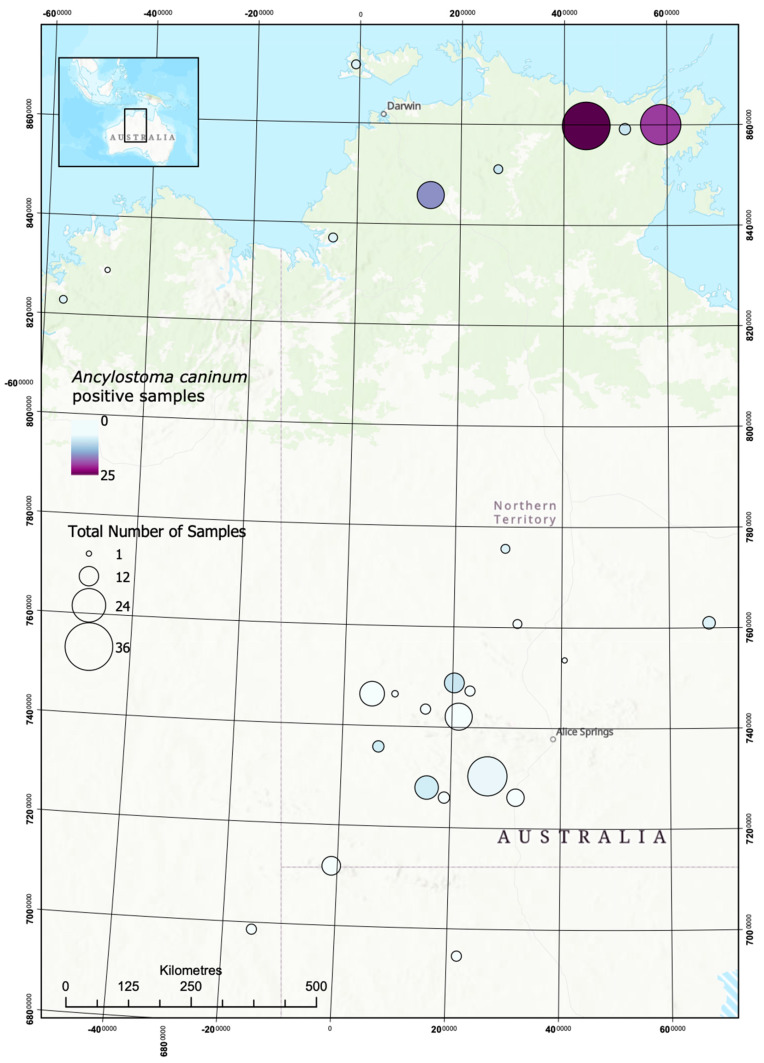
Opportunistic mapping of *A. cacinum* in dogs in remote communities.

Maps showing sample locations and *S. stercoralis* and hookworm prevalence in dogs are shown in Figure 2 and Figure 3. 

### 2.3. Association of Hookworms with Strongyloidiasis

Chi-squared analysis did not identify a statistically significant association between *S. stercoralis* and *A. caninum* (*x^2^* (1) = 0.003, *p* = 0.958, *n* = 274). Of the 274 dog faecal samples, 6.9% (19/274) tested positive for both *S. stercoralis* and *A. caninum* and 53.6% tested negative for both parasites (Figure 1).

None of the non-dog faecal samples were infected with *S. stercoralis* or *A. caninum.*

## 3. Discussion

In this study, we used the quantitative polymerase chain reaction (qPCR) technique to detect potentially zoonotic *S. stercoralis* and zoonotic hookworms in dog faecal samples collected from remote communities in Northern and Central Australia. The prevalence of *S. stercoralis* and *A. caninum* in dogs was found to be high. All samples were negative for *A. ceylanicum*, *A. braziliense* and *U. stenocephala*, which supports previous studies demonstrating that *A. caninum* is the most common hookworm in dogs living in remote communities in Australia [15,33,34].

*Ancylostoma caninum* is known to cause eosinophilic enteritis in humans. Although infection is asymptomatic in most cases, symptoms can include strong abdominal pain with or without peripheral eosinophilia, nausea, diarrhoea, anorexia and allergic reactions [34]. In cases in which the infection is patent, the impact of a hookworm infection on nutritional status and immunocompetence may be associated with other health problems, including increased susceptibility to other helminth infections [36].

Although *A. ceylanicum* is the predominant hookworm affecting dogs and cats in Asia, it was reported only recently in dogs in Australia [28]. However, its presence in a cat from far north Queensland was retrospectively dated back to 1994 [37]. *Ancylostoma ceylanicum* was detected for the first time in Australia in 6.5% of dogs from rural and urban areas in Broome, Brisbane, the Sunshine Coast, Melbourne and Alice Springs [15]. More recently, *A. caninum* and *A. ceylanicum* infections were reported for the first time at a prevalence of 98.4% (62/63) and 1.6% (1/64), respectively, in domestic dogs in far north Queensland [24]. The same study discovered prevalence of *A. ceylanicum* ranging from 25% to 100% in the soil in different communities in far north Queensland [24]. A study of dingoes and dogs in Northeast Queensland reported 100% (35/35) and 11% (4/35) prevalence of *A. caninum* and *A. ceylanicum*, respectively, in dingoes, and a 92% (78/85) prevalence of *A. caninum* in dogs, based on both necropsy and faecal examination [34]. A more recent study found 66% (93/141) of camp dogs in remote communities in Western Australia to be infected with *A. caninum*, based on molecular examination [33]. The absence of *A. ceylanicum* in this study was likely due to the climatic conditions, such as dry weather, of the study area at the time of sampling. To date, there has been no evidence that *A. ceylanicum* possesses the biological advantage of undergoing arrested development, a process in which larvae undergo a period of hypobiosis in host tissue and then resume development in the intestinal tract when climatic conditions favour transmission [38].

The absence of *U. stenocephala* in the samples is supported by its association with lower temperatures [39]. *U. stenocephala* is predominately found in the southern regions of Australia, because the optimum temperature conditions for *U. stenocephala* larvae development up to the infective stage is between 7.5 and 27 °C and the ideal temperature for the free-living stages is 20 °C [40]. Likewise, previous studies exclusively detected *A. braziliense* in dogs located in North Queensland [15,41].

Molecular detection methods have been shown to be highly effective in the detection of *S. stercoralis* and hookworms in faecal samples [42,43,44,45,46]. However, the sensitivity of the PCR technique for the detection of *S. stercoralis* is lower when there is a low number of larvae in the faeces [47]. Although the S. *stercoralis* primers and probe used in this study have been described as *S. stercoralis*-specific [43,47], they can also amplify *S. ratti*, as previously demonstrated [47], meaning that for environmental samples we can only assume that positive samples contain *Strongyloides* spp. As for the dogs, we know from the previous genotyping study on dogs living in remote communities in Australia that dogs have been found to be infected with *S. stercoralis* strains [12]. However, due to the possibility that dogs engaged in hunting and coprophagia, we cannot rule out the possibility of mechanical ingestion of other species of *Strongyloides*, including human-sourced species.

Increased humidity and temperature are typically associated with the presence of *Ancylostoma* spp. and *S. stercoralis.* Tropical climates have been shown to be associated with multiple parasite infections in humans [36,48]. Hookworm infection intensity has also been associated with multiparasitism because co-infection with hookworms weakens the immune system of the host. The intensity of a strongyloidiasis infection is in turn highly dependent on the immune status of the host [36,48]. This illustrates the importance of detecting and differentiating parasite infections. Moreover, the indiscriminate use of anthelmintic drugs may cause the development of anthelmintic resistance [49]. A study conducted in Brazil showed a strong association between hookworm and other helminth infections (but not *S. stercoralis* infections) in humans [36]. In the present study, we did not find any significant association between *Strongyloides* spp. and *A. caninum* in dogs. Non-infected dogs might be a result of dog health programs targeted at desexing and deworming dogs in remote communities, which are administered by the Animal Management in Rural and Remote Indigenous Communities (AMRRIC) organisation.

Infections of both *S. stercoralis* and hookworm occur through exposure to soil contaminated with free-living infective stages of a parasite [1]. In the studied locations, dogs live in close proximity to their owners. Climate, sanitation, hygiene, environmental contamination with human or dog faeces and lack of knowledge of STH diseases are the main factors influencing the persistence of the disease, and can also influence its transmission [5,32,50]. Our findings demonstrate the importance of the “One Health” initiative, an approach which considers veterinary and public health interventions together. The One Health approach should be central to the development of methods of eliminating *S. stercoralis* and hookworms. To maintain the health of both dogs and humans, veterinarians and pet owners are encouraged to coordinate their efforts and to work in partnership [51].

The findings of this study need to be interpreted in light of its limitations. The faeces samples were collected from the ground, rather than directly from the rectums of dogs. Therefore, some of the samples that were collected were found not to be from dogs. Samples collected from the environment might have been contaminated with DNA from extraneous environmental organisms, which could have caused further inhibition of the DNA of the target organisms (*Strongyloides* spp. and hookworms) [52,53]. Researchers could also have accidentally collected faeces that were old enough for parasites’ DNA to have degraded. Both these limitations could have resulted in false negatives. Furthermore, the possibility that the dogs had engaged in hunting and coprophagia could lead to false positives. The opportunistic sampling method did not allow us to consider risk factors associated with parasite prevalence, such as seasonal variation, climate conditions or the use of anthelmintic drugs. Furthermore, there was significant variation in the number of samples from each geographical area.

The aim of this study was to map the prevalence of *S. stercoralis* and hookworm infection in dogs in remote communities in Australia based on the molecular screening of dog faeces. The objective was to develop and optimise detection methods that can be applied in similar environmental settings without laboratory facilities and in a respectful and non-intrusive manner. We detected high levels of *S. stercoralis* and *A. caninum* in dog faecal samples collected from remote communities. Future research is needed to examine parasite prevalence in both dogs and humans from the same communities to determine whether there is an association between them, and thus to assess the zoonotic potential of dogs to transmit the diseases. Given the zoonotic nature of these parasitic species, the findings of this study can be used to develop control measures to maintain dog and human health.

## 4. Materials and Methods

### 4.1. Ethical Considerations

The project was registered with the Flinders University Animal Welfare Committee, part of the Research Development and Support division. The research was approved by the Social and Behavioural Research Ethics Committee (SBREC) (No. 6852, dated 1 June, 2015). For dog faeces collected from residential or private land, consent was obtained from the owners of the dogs or from the local managers of the communities.

### 4.2. Study Area and Population

Two hundred and eighty-five faecal samples presumed to be from dogs were collected from remote communities across the Northern Territory, Central Australia, Western Australia, and the northwest of South Australia during 2016 and 2019. The samples were collected from 27 locations in total, including 23 communities in the Northern Territory, two communities in the northern parts of Western Australia, one community in the northwest of South Australia and in the vicinity of Alice Springs.

### 4.3. Specimen Collection and DNA Extraction

Faeces were collected either by the Flinders University researchers, Northern Territory Department of Health environmental health officers (EHOs) or veterinarians primarily from the AMRRIC organisation.

In the cases where samples were collected by EHOs or representatives of AMRRIC, they would do so during their routine inspections or dog treatments. A sampling package containing the project’s information sheet, risk assessment and consent forms, sampling instructions and sampling equipment was provided to them in advance.

Permission from the community elders, Traditional Owners or community managers was obtained prior to collecting samples from private or residential land. Approximately 2–3 g of faeces were collected and preserved immediately in 6 mL DESS (dimethyl sulfoxide, disodium EDTA, and saturated NaCl) and kept at room temperature [54]. The samples were shipped to the Environmental Health laboratory, Flinders University, within 30 days after collection for further sample processing. The genomic DNA was extracted using the PowerSoil DNA Isolation Kit (QIAGEN, Hilden, Germany) as described previously [12,47].

### 4.4. Real-Time PCR Assays

The real-time PCR assay was adopted from Verweij et al. [43] using *S. stercoralis*-specific primers (Stro18S-1530F and Stro18S-1630R) and a probe (Stro18S-1586T) targeting the 101 base pair (bp) region of the 18S rRNA, and conducted as described previously [12]. All qPCR reactions were performed in triplicate on the two-channel Corbett Rotor-Gene 6000 machine (QIAGEN, Hilden, Germany). The primers, probes and qPCR conditions are shown in Table 1. It should be noted that although this primer/probe set is considered specific for *S. stercoralis*, it can also amplify other species of *Strongyloides,* including *Strongyloides ratti*.

Positive, non-template and negative control samples were included in each qPCR run. The cycle quantification (Cq) value for *S. stercoralis* was 0.02 to 0.03. A sample was considered positive when the cycle threshold (Ct) value was lower than the mean negative Ct value minus 2.6 standard deviations of a mean negative control Ct value [54]. Positive samples were amplified in every qPCR reaction.

Multiplex qPCR assays for detection of *A. ceylanicum, A. caninum, A. braziliense* and *U. stenocephala* using primers and probes targeting the internal transcribed spacer 1 (*ITS1*) gene were adopted and performed as described by Massetti et al. [46].

Synthetic block gene fragments (IDT Technologies, Skokie, Illinois, USA) of *ITS1* genes targeted by the PCR primers and probes for *A. ceylanicum, A. caninum, A. braziliense* and *U. stenocephala* were used as positive controls in the PCR runs (Table 2). Nuclease-free water was used as the non-template or negative control. Synthetic block gene fragments (IDT Technologies, Skokie, Illinois, USA) of a herpes virus (Equine herpesvirus type 4, accession number KT324745.1) was used as an internal control. Primers and a probe to amplify a region of the dog mtDNA (*Canis lupus familiaris or Canis lupus dingo,* accession numbers MH 105047.1 and MH035676.1) were used as DNA extraction controls in all runs. Primers, probes and qPCR conditions are shown in Table 1. The GenBank Accession numbers and sequences of the synthetic block gene fragments used as controls in this study are presented in Table 2. All hookworm qPCR reactions were performed in duplicate on the multiplex channel Corbett Rotor-Gene 6000 machine (QIAGEN, Hilden, Germany).

The Cq value for *A. ceylanicum* and *A. caninum* was 0.05 and a Ct value of 32 was established. The Cq values for *A. braziliense* and *U. stenocephala* were 0.08 and 0.1, respectively, and the Ct value was set to 32.

Synthetic block gene fragments of hookworms were also spiked with negative dog DNA and analysed with qPCR to check for any inhibitors that might be contained in dog DNA. All spiked hookworm synthetic block gene fragments were amplified by means of qPCR.

### 4.5. Statistical Analysis

A chi-square independence test was performed to determine whether there was an association between hookworms and *S. stercoralis* infection. Data were analysed using Statistical Package for Social Sciences (SPSS) software (SPSS for Windows, Version 23, IBM) and Excel 2016 (Microsoft).

## Figures and Tables

**Table 1:** Primers and probes and PCR conditions.

Primer/Probe	Amplicon	Sequence	Reaction Conditions
Stro18S-1530F Stro18S-1630R Stro18S-1586T FAM	rDNA 101 bp	5′-GAATTCCAAGTAAACGTAAGTCATTAGC-3′ 5′-TGCCTCTGGATATTGCTCAGTTC-3′ 5′-FAM-ACACACCGGCCGTCGCTGC-3′-BHQ1	**Step 1:** 95 °C for 15 min, **Step 2:** 95 °C for 15 s, **Step 3:** 60 °C for 30 s. Repeat steps two and three 40 times.
*A. cancey* F *A. cancey* R Ahumanceylanicum probe Acantub probe	ITS1 region	5′- GGGAAGGTTGGGAGTATCG-3′ 5′- CGAACTTCGCACAGCAATC-3′ 5′- Cy5/CCGTTC+CTGGGTGGC/3IABkRQSp/-3′ 5′-HEX/ AG+T+CGT+T+A+C+TGG/3IABkRFQ/-3′	**Step 1:** 95 °C for 2 min, **Step 2:** 95 °C for 15 s, **Step 3:** 60°C for 60 s. Repeat steps two and three 40 times.
Uncbraz F Uncbraz R Unc Probe Abra probe	ITS1 region	5′- GAG CTT TAG ACT TGA TGA GCA TTG-3′ 5′- GCA GAT CAT TAA GGT TTC CTG AC-3′ 5’-/5HEX/CAT TAG GCG /ZEN/GCA ACG TCT GGT G/3IABkFQ/-3′ 5’-/56FAM/TGA GCG CTA /ZEN/GGC TAA CGC CT/3IABkFQ/-3’	**Step 1:** 95 °C for 2 min, **Step 2:** 95 °C for 15 s, **Step 3:** 64 °C for 60 s. Repeat steps two and three 40 times.
EMV F ENV R ENV probe	Equine herpesvirus type 4	5′-GATGACACTAGCG-ACTTCGA-3′ 5′-CAGGGCAGAAACC-ATAGACA-3′ 5′-TEX-TTTCGCGTGC-CTCCTCCAG-IBRQ-3′	**Step 1:** 95 °C for 2 min, **Step 2:** 95 °C for 15 s, **Step 3:** 60 °C for 60 s. Repeat steps two and three 40 times.
Dog F Dog R Dog probe	mtDNA	5′-CGACCTCGATGTTGGATCAG-3′ 5′-GAACTCAGATCACGTAGGACTTT-3′ 5′-FAM/ CCTAATGGT/ ZEN/ GCAGCAGCTATTAA/ LABKFQ-3′	**Step 1:** 95 °C for 2 min, **Step 2:** 95 °C for 15 s, **Step 3:** 60 °C for 60 s. Repeat steps two and three 40 times.

**Table 2:** Synthetic block gene fragments used for positive controls.

Species	GenBank Accession Number	Sequence
*Ancylostoma ceylanicum*	DQ780009.1	CGTGCTAGTCTTCAGGACTTTGTCGGGAAGGTTGGGAGTATCGCCCCCCGTTACAGCCCTACGTGAGGTGTCTATGTGCAGCAAGAGCCGTTCCTGGGTGGCGGCAGTGATTGCTGTGCGAAGTTCGCGTTTCGCTGAGCTTTAGACTTGAG
*Ancylostoma duodenale/Ancylostoma caninum*	EU344797.1	CGTGCTAGTCTTCACGACTTTGTCGGGAAGGTTGGGAGTATCGCCCCCCGTTATAGCCCTACGTAAGGTGTCTATGTGCAGCAAGAGTCGTTACTGGGTGACGGCAGTGATTGCTGTGCGAAGTTCGCGTTTCGCTGAGCTTTAGACTTGAT
*Ancylostoma braziliense*	JQ812692.1	TGTACGAAGCTCGCGGTTTCGTCAGAGCTTTAGACTTGATGAGCATTGCTAGAATGCCGCCTTACCTGCTTGTGTTGGTGGTTGAGCGCTAGGCTAACGCCTGGTGCGGCACCTGTCTGTCAGGAAACCTTAATGATCTGCTAACGCGGACGCCAGCACAGCAAT
*Uncinaria stenocephala*	HQ262054.1	GCTGTGCGAAGTTCGCGTTTCGCTGAGCTTTAGACTTGATGAGCATTGCTGGAATGCCGCCTTACTGTTTGTGTTGGTGGTTGGGCATTAGGCGGCAACGTCTGGTGCGACACCTGTTTGTCAGGAAACCTTAATGATCTGCTCACGTGGACGCCAATACAGCACT
*Equid herpesvirus*	KT324745.1	ATGAAAGCTCTATACCCAATAACAACCAGGAGCCTTAAAAACAAAGCCAAAGCCTCATACGGCCAAAACGACGATGATGACACTAGCGACTTCGATGAAGCCAAGCTGGAGGAGGCACGCGAAATGATCAAATATATGTCTATGGTTTCTGCCCTGGAAAAACAGGAAAAAAAGGCAATGAAGAAAAACAAGGGGGTTGGACTTATTGCC
